# Robotic total knee arthroplasty for moderate to high-grade valgus knee deformity: technique and outcomes

**DOI:** 10.1051/sicotj/2025005

**Published:** 2025-03-04

**Authors:** Kanukuntla Kalyan, Ashish Singh, Purushotam Kumar, Akash Chandrashekar Gundalli, Sudhir Shankar Mane, Himanshu Swarnkar, Lavanya Singh

**Affiliations:** 1 Anup Institute of Orthopaedics & Rehabilitation G75-77, PC Colony Kankarbagh, Patna Bihar 800020 India; 2 The Hazeley Academy Emperor Dr Hazeley Milton Keynes MK8 0PT United Kingdom

**Keywords:** Robotic arm-assisted total knee arthroplasty, Valgus deformity, Predictive gap balancing, Functional alignment

## Abstract

*Introduction*: Although the surgical techniques and functional outcomes of conventional total knee arthroplasty (TKA) are well-established, there is limited data available on robotic arm-assisted TKA (RATKA) in the context of valgus knee arthroplasty. The purpose of this study is to assess the efficacy of RATKA in the correction of moderate to severe valgus knee deformities using minimally constrained implants and to evaluate the short-term functional outcomes associated with this technique. *Methods*: This prospective study was conducted on patients with moderate to severe grade valgus knee deformity who underwent RATKA from August 1, 2020 to May 31, 2022. Of 873 primary RATKA cases, 48 cases had valgus knee deformities. Among these, 27 had grade 2–3 valgus with intact medial collateral ligament (MCL), two had grade 3 valgus with incompetent MCL, 14 had grade 1 valgus, and five had post-traumatic valgus deformities. Over a two-year follow-up period, functional outcomes were assessed using the Western Ontario and McMaster Universities Osteoarthritis Index (WOMAC) and Knee Society Score (KSS), and complications were documented; however, radiological outcomes were not analyzed. *Results*: Among 27 patients with Grade 2–3 valgus, the final cohort included 21 patients (24 knees). The mean age was 58.33 ± 9.63 years and 70.8% were female. Ten (41.7%) patients had rheumatoid arthritis and 14 (58.3%) had degenerative osteoarthritis (OA). The median surgical time was 68.00 (13.00) minutes, and the median blood loss was 478.45 (176.25) mL. The valgus grade was reduced from a baseline value of 22.43 ± 7.05 degrees to 5.26 ± 1.53 degrees at 6 weeks. The WOMAC scores improved from 67.58 ± 7.27 at baseline to 1.38 ± 0.57 in the second year post-operatively. Similarly, the KSS scores improved from 26.67 ± 10.34 at baseline to 181.96 ± 7.20 in the second year. One patient sustained a Type II supracondylar femur fracture after a fall, managed with distal femur arthroplasty, while another had delayed tibia pin tract healing, treated with antibiotics and dressings. *Conclusion*: RATKA facilitates precise correction of moderate to severe valgus deformity through enhanced surgical planning and execution, achieving adequate functional outcomes with minimal complications through the application of functional alignment philosophy.

## Introduction

Valgus deformity of the knee is defined when the arithmetic hip-knee ankle angle is greater than +2° [1]. Though the most common cause of knee valgus deformity is primary osteoarthritis, which constitutes 10% of total knee arthroplasty (TKA) cases [2], secondary causes are not uncommon [3].

Valgus deformity of the knee involves intra-articular and extra-articular factors including hypoplastic lateral femur condyle, lateral tibial plateau deformity, external rotation deformity, metaphyseal remodeling, and patellar malalignment [4]. Tightening of the lateral soft tissue structures can involve the iliotibial band, the lateral collateral ligament, the posterolateral corner, the posterior cruciate ligament (PCL), the popliteus tendon, and the lateral head of the gastrocnemius [5]. These soft tissue contractures are responsible for lateral patellar subluxation, patellofemoral mal-tracking, and post-operative knee instability. Tightening of lateral soft tissue structures may result in attenuation of the medial collateral ligament (MCL), particularly in severe deformity [6]. In these instances, achieving a well-balanced TKA can be challenging.

Valgus deformity of the knee is classified in different ways, typically based on the severity of the valgus malalignment and the involvement of the soft tissues. Ranawat et al. [2] classified valgus deformity of the knee into three grades: grade I, grade II, and grade III. In a grade I case, the mechanical axis deviation is less than 10°, correctable with an intact MCL. With grade II, the axis of deviation ranges from 10° to 20° with a functionally elongated MCL. In grade III, the deviation exceeds 20° accompanied by severe impairment of medial stabilizing elements where constrained implant may be necessary. Grade I, II, and III deformities constitute 80%, 15%, and 5% of cases. Grade III deformity constitutes only 0.5% of patients undergoing TKA [7].

Conventional TKA, performed without the assistance of robotic systems, has long been the standard approach for managing moderate to severe valgus deformity. Although it has been in practice for several decades, several technical challenges are still being faced by even the most experienced surgeons. Common challenges include addressing the soft tissues, i.e., MCL being intact or incompetent [8] and the lateral soft tissue extensive release [9], the approach (medial or lateral parapatellar) [10, 11], the difficulty in inserting the intramedullary femur guide, the use of anterior or posterior referencing in lateral condylar hypoplasia [12], under or overcorrection of the deformity [13], restoration of the joint line [7], patellar tracking [14], extensive bone cuts to achieve a rectangular gap [15], the balancing of the femoral flexion gap with femur component rotation [16], the use of lateral femoral sliding osteotomy for rigid valgus deformities [17], the use of higher Polyethylene insert, and the need of constrained condylar knee (CCK) or hinged implants [18]. In the context of constrained implants, cruciate-retaining (CR) and cruciate-substituting (CS) are unconstrained whereas posterior stabilized (PS) implants are partially constrained [19]. However, Alesi et al. [20] reported that there is no sufficient evidence in the literature regarding the optimal technique for managing valgus deformity, including lateral soft tissue release. The present study aimed to address the efficacy, short-term outcome, surgical challenges, and complications associated with robotic arm-assisted technology in correcting moderate to severe valgus knee deformity with minimally constrained implants.

## Materials and methods

### Study design, settings, and patient selection

This was a prospective study conducted on patients with valgus knee deformity and symptomatic osteoarthritis who underwent Primary Robotic Total Knee Arthroplasty in our hospital from August 1, 2020, to May 31, 2022, with 2 years of post-operative follow-up. A total of 873 primary RATKA were done during this period, of which 48 patients had valgus knee deformity and the remaining had knees with varus deformity. Of these 48 patients, 27 had grade 2 and 3 valgus deformity [2] with intact MCL, 2 patients had grade 3 valgus deformity with incompetent MCL, 14 patients had grade 1 valgus deformity and 5 had post-traumatic valgus deformity. The final cohort included 27 patients with grade 2 and 3 valgus deformity.

This study was conducted after ethical committee clearance (protocol number 2020/03TKAVD). Written informed consent was obtained from all the patients. Patients with grade 2 and 3 deformities with intact MCL were included in the study. Patients with Grade 1 deformity, incompetent MCL, post-traumatic osteoarthritis, and those who were lost to follow-up were excluded from this study. Six patients were lost to follow-up. All recruited patients underwent TKA using MAKO^®^ arm-assisted robotic technology (RIO; Robotic Arm Interactive Orthopedic, MAKO Stryker, Fort Lauderdale, Florida), and a single MAKO-certified arthroplasty surgeon performed all surgeries through the midline medial parapatellar approach. Any complications in the follow-up period were documented. Radiological outcomes were not analyzed. Complications were classified as major, requiring further surgical intervention, and minor, not necessitating additional surgery [21].

### Pre-operative planning

#### Clinical evaluation

**Figure 1:**
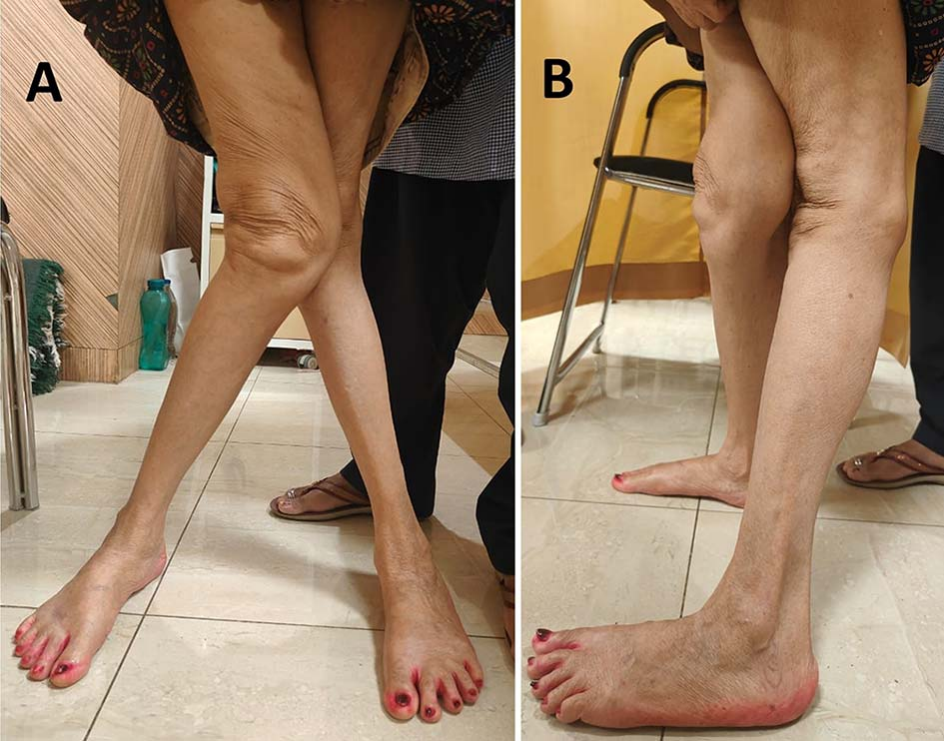
Pre-operative image of a patient showing severe knee valgus deformity of both knees.

All patients were clinically examined for knee deformity in the coronal and sagittal plane, range of motion (ROM), anteroposterior and mediolateral instability, and gait (Figure 1) to assess dynamic instabilities. The medial collateral ligament was assessed on whether it was intact or incompetent.

#### Radiographic planning

**Figure 2:**
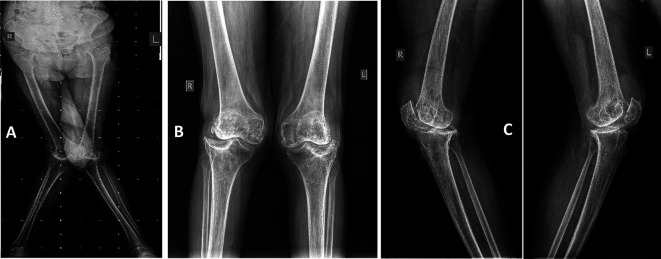
(A) Pre-operative bilateral lower limb scanogram, (B) Anteroposterior view of both knees, (C) Lateral view of both knees. Note the hypoplasia of lateral femoral condyle and the deformity in the tibial plateau.

All patients were subjected to a full lower limb standing scanogram [22] (Figure 2) including bilateral weight-bearing anteroposterior and lateral views, with patella skyline views. Pre-operative computed tomography (CT) scans of the knee with hip and ankle sections were taken.

### Surgical technique

#### MAKO 1.0 Robotic TKA pre-operative planning

MAKO SmartRobotics^TM^ combines three key components into one platform: 3-dimensional (3D) CT-based planning, AccuStop^TM^ haptic technology, and insightful data analytics [23]. MAKO^®^ or the robotic arm interactive orthopedic system, is a semi-active, CT-based, closed platform system that was approved by the FDA in 2017. The MAKO 1.0 platform works on the functional alignment philosophy. The goals and boundaries of pre-operative planning include quantifying the inherent knee deformity, evaluating bone defects, determining appropriate implant sizing and positioning, depth of bone resection, and tibial slope needed for deformity correction and optimal implant positioning. The primary goal is to restore the joint’s obliquity and alignment to match the natural orientation dictated by the soft tissues, thereby optimizing knee function and kinematics.

#### Alignment philosophy

Functional alignment (FA) is an emerging philosophy that reconstructs 3D constitutional alignment while respecting the soft-tissue envelope [24]. FA aims to position implants in a 3D orientation that minimally disrupts the soft tissues, restoring the joint’s obliquity and plan according to ligament-driven anatomy. Several robotic platforms enable preemptive gap prediction by adjusting implant positioning before bone cuts, marking a significant shift from kinematic approaches that rely on measured resection [25]. In FA, balanced laxity is achieved before cuts are made by placing the implant in a position that fits the behavior of the patient’s knee through an arc of flexion.

Leveraging robotic platforms, FA enables precise adjustments to femoral and tibial cuts, implant placement, and soft-tissue balancing in three planes, achieving extension-flexion gaps and tension within 1.5 mm of each other and a maximum of 2 mm from the global implant thickness (which is 17.5 mm when a 9 mm polyethylene insert is used) [26]. This minimizes the need for extensive periarticular soft-tissue releases. FA provides an effective method for addressing valgus deformities. Robotic platforms enhance precision within 1° or 2°, reducing alignment outliers and improving the reproducibility of non-neutral alignment targets [27]. Final gap assessments are conducted post-implantation in full extension and 90° flexion, ensuring a balanced knee with minimal residual gaps. By tailoring implant size and position to individual anatomy and soft tissue behavior, FA respects variability and offers a precise approach to optimizing knee function and kinematics in TKA.

#### Surgical exposure

**Figure 3:**
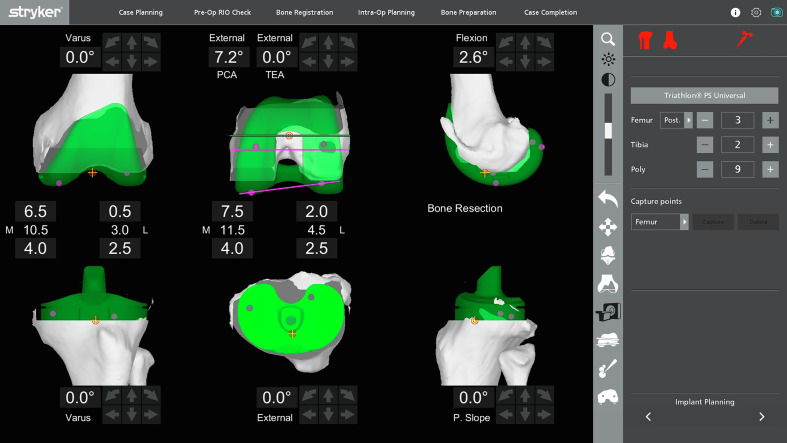
The native pre-operative plan set for a 0° of mechanical alignment, 0° of distal femoral and proximal tibial bone resections, and femoral rotation parallel to the femoral transepicondylar axis.

An Esmarch bandage and pneumatic tourniquet were used with a standard midline medial parapatellar approach for all patients. Two 4.0 mm threaded pins were inserted bicortically into the femur, 10 cm proximal to the patella, through stab incisions with the knee flexed to 90° to elongate the quadriceps muscle. Similarly, two 3.2 mm bicortical threaded pins were placed in the tibia, 10 cm distal to the joint line [21]. Arrays with trackers were mounted on the pins creating a stereo-tracking system between the patient and robotic console (Figure 3). The tibial checkpoint was positioned medial to the tibial tubercle, while the femoral checkpoint was placed proximal and anterior to the medial femoral epicondyle. To ensure stability, both checkpoints were secured on hard bone, away from the nearest bone cuts. The distal femur and the proximal tibia were registered to be linked to the CT-determined 3D virtual bone model [28]. Osteophytes on the patella were removed, and denervation was carried out circumferentially around the patella using electrocautery to a depth of 2–3 mm [29].

#### Gap balancing technique

**Figure 4:**
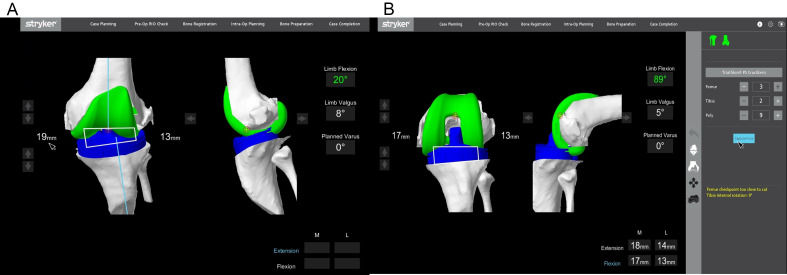
(A) Pose capture of the knee with varus stress applied to assess lateral soft tissue gap near full extension. (B) Pose capture of the knee in flexion with soft tissue tensioners.

In extension, medial and lateral gaps were measured with valgus and varus stress, respectively (Figure 4). Flexion gaps were then measured by placing the spacer spoons (knee tensioner) in 90° flexion in the medial and lateral tibiofemoral joint space.

#### Intraoperative modification of pre-operative plan

Intraoperatively, the femoral and tibia component positions were adjusted to balance the medial and lateral gaps, both in flexion and extension. This helped lessen the need for large soft tissue releases and bone cuts. In valgus knees, the lateral compartment is more contracted than the medial compartment. The aim was to achieve a rectangular gap for optimal placement of the implants. In this study, no soft tissue releases were performed. Intraoperative gaps in the medial and lateral compartments during flexion and extension were targeted at 18 mm to ensure balanced symmetry and minimize instability. This approach avoided complications from asymmetrical gaps, such as instability in flexion or hyperextension in extension, which can result from inconsistent gap balancing. Using the MAKO robotic platform, precise pre-operative adjustments ensured these targets were met intraoperatively, reducing variability. Equal gaps effectively maintained stability, particularly in valgus deformities with differing medial and lateral tension.

**Figure 5:**
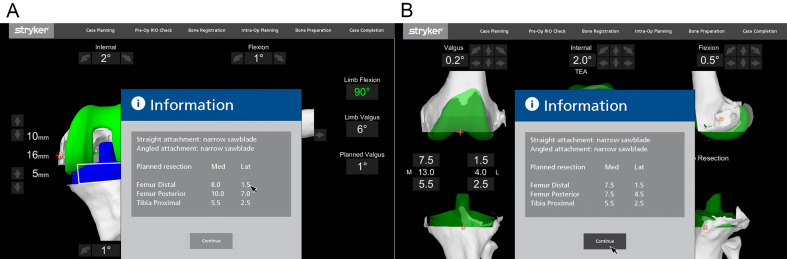
(A) Bone cuts after predictive gap balancing. The amount of bone cuts is precisely determined. (B) Decrease in posterior femur cuts after virtual adjustment of implant position.

For the planned resection of bony cuts (medial and lateral), the distal femur, posterior femur, and proximal tibia were noted on the monitor. The extent of bone cuts were quantified and could be modified by changing the component position to balance the joint line (Figure 5). Femoral component flexion and notching were checked, visualized, and adjusted accordingly. The femur and tibia component sizes were obtained during the pre-operative planning. Intra-operatively, they were verified with real-time data. MAKO 1.0 also allows pre-operative assessment and adjustment of parameters such as trochlear groove translation and over- or under-stuffing of the trochlea [24]. The posterior slope was set at 3° for all cruciate-retaining and 0° for posterior-stabilized implants.

In the patients with lateral femur condyle hypoplasia, the knee presented valgus deformity in extension and flexion. In these cases, the femoral component was internally rotated and posteriorized to minimize the lateral condyle bone cut to as much as 0.5 mm, which is not possible in conventional TKA. In patients with lateral tibial articular defect, a spacer spoon was placed in the defect, masking the valgus of tibial origin.

##### Bone cuts

**Figure 6:**
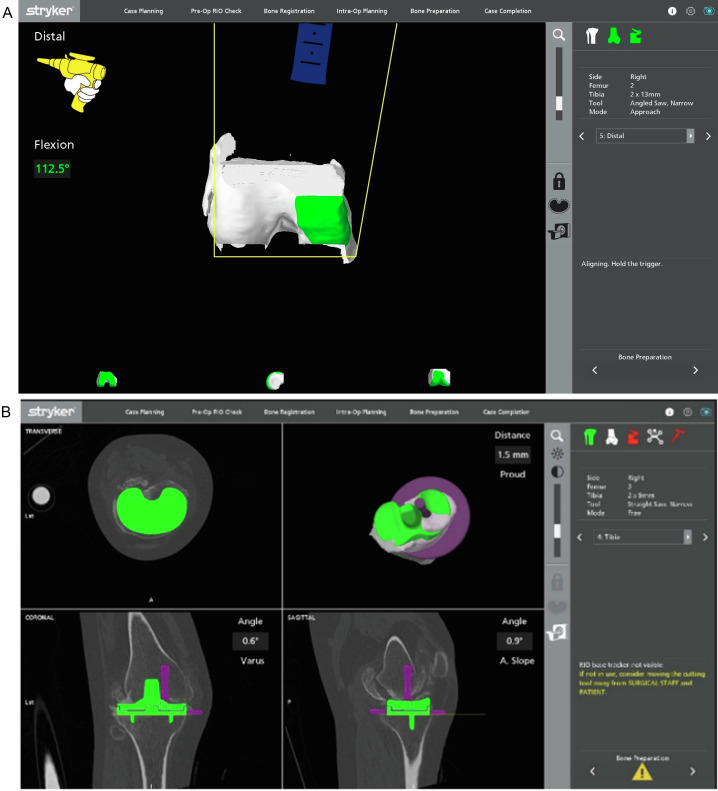
(A) As the saw completes the bone resection, the green area disappears. The green line represents the haptic boundary. The lateral condyle bone is preserved with a 0.5 mm cut. Note that the tip of the saw cannot migrate outside the haptic boundary. (B) The planar probe is used to check the accuracy of bone resections.

The robotic system shows the amount of bone resection in green color and turns to red if the bone cut is deeper than planned. The progression of the saw cut is noted by the disappearance of the green surface on the monitor. The alignment and depth accuracy of the saw cut can be verified with a planar probe (Figure 6).

##### Implantation

**Figure 7:**
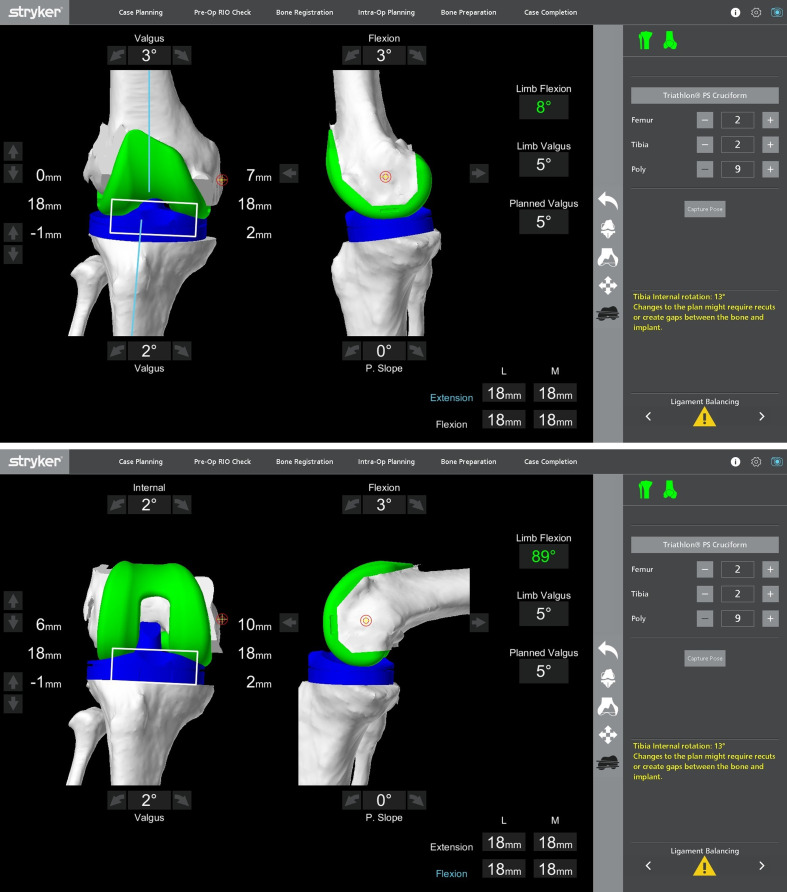
After placing trial implants, gaps were checked in extension and flexion to determine well-balanced mediolateral gaps in extension and flexion.

Quantified compartmental gap measurement in flexion and extension was visualized on the screen with the trial prosthesis [30]. The knee was moved through flexion and extension for patellar tracking, and adequate fitment of implants (Figure 7). Once a balanced knee and appropriate knee kinematics were confirmed, the definitive cruciate-retaining (CR), cruciate-substituting (CS), or posterior-stabilized (PS) femoral and tibial components (*Triathlon*® Stryker, Kalamazoo, MI, USA) were implanted with bone cement. In case of PS implants, a standard femoral box cut was made. In all patients, a drug cocktail comprising of 0.2% ropivacaine, tranexamic acid, and cefuroxime were locally infiltrated before closing the joint. Post-operatively, all patients received low molecular weight heparin and mechanical calf pumps for thromboprophylaxis. Post-operative rehabilitation followed the institution’s standardized integrated care plan.

### Clinical evaluation and functional outcomes

**Figure 8:**
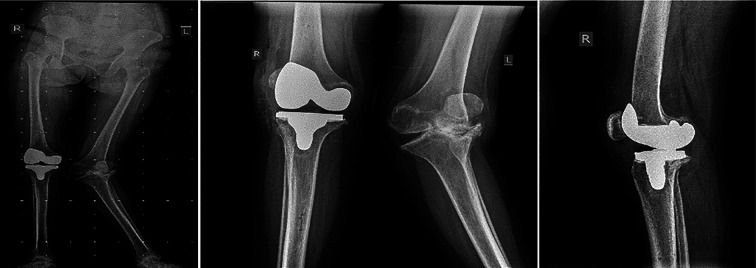
Post-operative X-ray of a unilaterally operated patient at 6 weeks, showing a bilateral lower limb scanogram along with standing anteroposterior and lateral views.

All 21 patients (24 knees) were followed-up for 24 months from August 1, 2020, to May 31, 2022. Patients were examined by specialized physiotherapists at different intervals; pre-operatively, post-operatively, day 5, week 6 (Figure 8), month 3, month 6, year 1, and year 2. During each visit, patients were assessed with the Knee Society Score (KSS), and the Western Ontario and McMaster Universities Osteoarthritis Index (WOMAC).

### Statistical analysis

The analysis was performed with IBM SPSS Statistics for Windows, version 23 (IBM Corp., Armonk, NY, USA). Categorical data were expressed using frequencies and percentages. Shapiro-Wilk test was used for checking normality in continuous data. Continuous parametric data (such as age, BMI, blood loss, and time) were reported in the form of mean and standard deviation (SD). Continuous non-parametric data were expressed as median (IQR) and Wilcoxon signed ranks (*Z*-test) were used for dependent samples to compare the data. Statistical significance was set at a probability level of 95% (*p* < 0.05).

## Results

During the study period, 21 patients (24 knees) with symptomatic knee osteoarthritis who underwent RATKA were recruited. The mean age of the recruited patients was 58.33 ± 9.63 years. Females constituted more than two-thirds (70.8%) of the sample. Approximately 58.3% of the sample had osteoarthritis, and 41.7% had rheumatoid arthritis. Valgus grade 2 constituted 45.8% of the sample, and grade 3, 54.2%. The average surgical time was 68.0 (13.0) minutes, and the mean volume of blood loss was 478.45 (176.25) mL, calculated by comparing the pre-operative and fifth-day post-operative hemoglobin levels [31]. Table 1 summarizes the demographic and baseline clinical data of the sample.

**Table 1:** Baseline clinical and radiological characteristics of the recruited patients (*n* = 21).

Variable	Normality	Descriptive statistics		
Age in years (Mean ± SD [min.–max.])	Normal	58.33 ± 9.63 [34.00–76.00]		
Sex (*n*, %)		Male		Female
		7 (29.2)		17 (70.8)
Diagnosis (*n*, %)		OA		RA
		14 (58.3)		10 (41.7)
Side (*n*, %)		Left		Right
		12 (50)		12 (50)
Valgus grade (*n*, %)		Grade 2		Grade 3
		11 (45.8)		13 (54.2)
BMI (Mean ± SD [min.–max.])	Normal	24.83 ± 3.05 [16.90–31.00] kg/m^2^		
BMI group (*n*, %)		Underweight	Normal	Overweight
		1 (4.2)	11 (45.8)	12 (50)
Blood loss (IQ (Median R) [min.–max.])	Non-normal	478.45 (176.25) [417.38–761.26] mL		
Surgical time in minutes (IQ (Median R) [min.–max.])	Non-normal	68.0 (13.0) [60.00–87.00]		

BMI: body mass index, max.: maximum, min.: minimum, *n*: number, OA: osteoarthritis, RA: rheumatoid arthritis, SD: standard deviation, IQR: interquartile range.

Table 2 details the characteristics of valgus deformity of the recruited sample. Of note, the mean valgus deformity grade was 20.94 (11.76) degrees at baseline, 6.03 (3.81) degrees at the fifth day post-operatively, and 5.09 (2.77) degrees at the sixth week post-operatively. The mean AG balancing for femur flexion, internal rotation, femur valgus, and tibia valgus were 2.08 ± 1.10, 200 (1.00), 2.00 (2.00), and 1.00 (1.00), respectively. For the WOMAC scores, there was a reduction from 67.58 ± 7.28 at baseline (pre-operatively) to 0.57 at year two (post-operatively). Similarly, there was an improvement in the KSS scores from 26.67 ± 10.34 at baseline (pre-operatively) to 181.96 ± 7.20 after 2 years (post-operatively).

**Table 2:** Characteristics of Type 2 and Type 3 valgus deformity of the recruited patients (*n* = 24).

Variable	Normality test	Minimum	Maximum	Mean	SD
				Median	IQR
Valgus grade					
Pre-operative valgus	Normal	11.13°	37.75°	22.43°	7.05°
				20.94°	11.76°
Post-operative valgus 5th day	Normal	2.38°	9.24°	5.83°	2.11°
				6.03°	3.81°
Post-operative valgus 6 weeks	Normal	2.98°	7.65°	5.26°	1.53°
				5.09°	2.77°
AG balancing					
Femur flexion	Normal	0.00°	4.00°	2.08°	1.10°
				2.00°	2.00°
Femur internal rotation	Non-normal	1.00°	3.00°	1.67°	0.64°
				2.00°	1.00°
Femur valgus	Non-normal	1.00°	3.00°	1.92°	0.77°
				2.00°	2.00°
Tiba valgus	Non-normal	0.00°	2.00°	1.25°	0.53°
				1.00°	1.00°
WOMAC					
Pre-operative	Normal	47°	79°	67.58°	7.28°
				67.50°	11.00°
Post-operative after 5 days	Normal	36°	68°	53.13°	8.13°
				52.50°	14.00°
Post-operative after 6 weeks	Normal	10°	47°	22.92°	10.55°
Post-operative after 3 months	Non-normal	5°	23°	11.04°	5.59°
				9.00°	10.0°
Post-operative after 6 months	Normal	2°	15°	7.62°	7.00°
				7.00°	3.85°
Post-operative after 1 year	Non-normal	1°	5°	2.67°	1.23°
				2.00°	2.00°
Post-operative after 2 years	Non-normal	1°	3°	1.38°	0.57°
				1.00°	1.00°
KSS					
Pre-operative	Normal	12°	52°	26.67°	10.34°
				25.50°	19.00°
Post-operative after 5 days	Normal	35°	76°	58.21°	12.61°
				59.50°	25.00°
Post-operative after 6 weeks	Normal	82°	138°	109.25°	18.50°
				110.50°	34.00°
Post-operative after 3 months	Normal	117°	167°	140.17°	14.76°
				139.00°	25.00°
Post-operative after 6 months	Normal	130°	177°	153.04°	12.14°
				150.00°	14.00°
Post-operative after 1 year	Non-normal	156°	182°	169.83°	8.85°
				172.50°	17.00°
Post-operative after 2 years	Normal	169°	196°	181.96°	7.20°
				183.00°	13.00°

AG: after gap, KSS: Knee Society Score, SD: standard deviation, WOMAC: Western Ontario and McMaster Universities Arthritis Index, IQR: interquartile range.

The valgus grades were compared between the different time points (Table 3). There was a significant improvement of the valgus grade between baseline (20.94, 11.76°) and the fifth day post-operatively (5.09, 2.77°) (*p* < 0.001). Similarly, the difference was significant between the valgus grade at baseline and week 6 post-operatively (*p* < 0.001). There was also a significant reduction of the valgus grade between the fifth day (6.03, 3.81°) and the sixth week (5.09, 2.77 ± 1.53) (*p* < 0.05).

**Table 3:** Comparison of valgus grades at different time points (*n* = 24).

	Timepoint 1	Timepoint 2	*Z*-value	*P*-value
Pre-operative versus post-operative day 5	Pre-operative	At day 5	−4.28	<0.001*
	20.94° (11.76)	6.03° (3.81)		
	(11.13–37.75)	(2.38–9.24)		
Pre-operative versus post-operative week 6	Pre-operative	At week 6	−4.29	<0.001*
	20.94° (11.76)	5.09° (2.77)		
	(11.13–37.75)	(2.98–7.65)		
Post-operative day 5 versus week 6	At day 5	At week 6	−2.48	0.013*
	6.03° (3.81)	5.09° (2.77)		
	(2.38–9.24)	(2.98–7.65)		

Descriptive statistics were presented as the median (interquartile range, IQR). The Wilcoxon signed-rank test (*Z*-test) was used to compare the outcomes, with a *p*-value of <0.005 considered statistically significant.

Concerning the gaps (Table 4), there was a significant reduction of the medial gap in extension from 20.00 (4.00) mm at baseline to 19 (1.0) mm post-operatively (*p* < 0.05). For the lateral extension, there was a significant increase in the gap from 15.00 (1.75) mm at baseline to 18.17 (0.00) mm post-operatively (*p* < 0.001). In flexion, there was a significant increase in the gap in lateral position only [from 14.00 (2.75) to 18.00 (0.00) (*p* < 0.001)]. However, the small change in the medial flexion gap was found to be statistically significant (*p* < 0.05).

**Table 4:** Comparison of pre-operative and post-operative gaps (in mm) in the recruited patients (*n* = 24).

Comparison of gap	Median	IQR	*Z*-statistic	*P*-value
Extension				
Medial				
Pre-operative	20.00	4.00	−2.96	0.003*
Post-operative	19.00	1.00		
Lateral				
Pre-operative	15.00	1.75	−4.33	<0.001*
Post-operative	18.17	0.00		
Flexion				
Medial				
Pre-operative	18.00	2.00	−2.00	0.045*
Post-operative	18.00	1.00		
Lateral				
Pre-operative	14.00	2.75	−4.309	<0.001*
Post-operative	18.00	0.00		

Descriptive statistics were presented as the median (interquartile range, IQR). The Wilcoxon signed-rank test (*Z*-test) was used to compare the outcomes, with a *p*-value of <0.005 considered statistically significant.

### Complications

One patient sustained a type II [32] supracondylar femur fracture 1 year after surgery due to a fall, which was ultimately managed with distal femur arthroplasty. Another patient had delayed healing of a tibia pin tract which was managed with a short antibiotic course and dressing. In this study, there were no other complications, such as thromboembolism, intraoperative fracture, peroneal nerve palsy, joint instability or loosening, patellofemoral maltracking, joint stiffness, septic arthritis, or RATKA abortion.

## Discussion

In this study, we aimed to assess the efficacy of RATKA in correcting moderate to severe valgus deformity of the knee and address the surgical technique and functional outcome. In conventional TKA, several surgical techniques were described to correct moderate and severe valgus deformity. However, there has been no consensus on the appropriate surgical technique to utilize, resulting in a wide variety of functional outcomes [33]. For instance, there is significant post-operative instability of approximately 24% [34] following considerable soft tissue release, which leads to higher morbidity. When instability cannot be controlled with balancing techniques alone, the necessity for constrained implants emerges [35].

### Alignment strategies in valgus knee deformities

Severe valgus knee deformities pose distinct challenges in TKA, necessitating careful alignment strategies to optimize outcomes. While neutral alignment has traditionally been thought to enhance implant longevity, long-term studies, such as those by Parratte et al., have challenged this notion, revealing no significant difference in survival rates between neutrally aligned TKAs and alignment outliers [36, 37].

To tackle these challenges, alignment strategies have evolved. In the 1980s, Hungerford and Krackow introduced anatomical alignment to enhance knee functionality by mimicking native knee alignment, which laid the foundation for the development of personalized alignment techniques [38]. These techniques include kinematic, inverse kinematic, restricted kinematic [39], and functional alignment [40]. Functional alignment (FA), in particular, aims to restore the natural joint orientation by positioning components in a way that minimally disrupts the soft-tissue envelope, incorporating coronal, rotational, and sagittal adjustments. Robotic platforms are crucial in FA, enabling virtual implant positioning and reducing the need for soft-tissue releases during surgery [41, 42].

For valgus knees, the literature highlights different approaches depending on the severity of the deformity. In cases of mild valgus deformities, correcting to neutral alignment results in better functional outcomes, higher patient satisfaction, and fewer complications than leaving a residual valgus [36]. In contrast, for severe valgus deformities, maintaining a moderate residual valgus (184°–189°) post-operatively has shown high satisfaction and functional outcomes without an increased risk of complications compared to neutral alignment [14, 43]. Additionally, studies confirm that residual valgus alignment in severe deformities does not increase the likelihood of revision surgery [36].

Despite advances in TKA, up to 50% of patients report ongoing symptoms, with more than 15% experiencing clinically significant patellofemoral dysfunction even when the patella is resurfaced [44]. These issues highlight the need to restore constitutional trochlear anatomy to achieve physiologic patellofemoral kinematics.

Alignment philosophy significantly influences trochlear groove recreation. While kinematic alignment places the femoral component unsafely in over 13% of cases, functional alignment most effectively restores trochlear depth across all flexion positions. The patella’s behavior, dictated by trochlear anatomy, plays a critical role in extensor mechanism function post-TKA [45].

Functional alignment addresses this by considering soft tissue laxity in flexion and extension, adjusting implant positions within defined boundaries to balance compartments while restoring the native trochlear groove [46].

#### Robotic arm-assisted technology in valgus knee TKA

RATKA has been offered as a technique for improving component position and soft tissue balancing in TKA [47]. These systems enable the dynamic and continuous assessment of medial and lateral gaps across the whole range of motion [48]. Rossi et al., in their study, found that a robotic system is useful for selecting the appropriate level of constraint by quantifying gaps and effectively addressing knee deformities [49]. In our study, RATKA proved effective in quantifying the medial and lateral compartment gaps during flexion and extension. Based on these assessments and the competency of the PCL, CR, CS, or PS implants were selected accordingly. Shatrov et al. have elaborated the guidelines for implementing the functional alignment philosophy in the valgus morphotype [26].

RATKA was found to achieve a significant improvement in correcting valgus deformity and gap balancing. In our study, we achieved deformity correction from 22.43 ± 7.05° to 5.26 ± 1.53 with no soft tissue releases and Cruciate retaining or Posterior stabilized implants, which is one of the recognized TKA standards [50]. In a study by Pagoti et al., the mean coronal alignment improved from 9.6° (±2.3) at 1 year to 5.6° (±2.7) at 45.7 months, with the focus on achieving a balanced extension gap rather than a neutral mechanical axis [51]. A valgus alignment of up to 7° was considered acceptable, and patients were generally unconcerned about the residual deformity, likely due to their pre-existing valgus alignment. Similarly, in our study, the mean valgus deformity improved from 22.43° (±7.05) pre-operatively to 5.83° (±2.11) on day five and 5.26° (±1.53) at 6 weeks post-operatively. Parratte et al. suggested that targeting a neutral mechanical axis may not always correlate with long-term success in TKA [37].

This is comparable to findings from previous research which showed how RATKA was able to reduce alignment outliers and improve gap balancing post-operatively when compared to conventional TKA [52]. In conventional TKA, balancing gaps in flexion requires anterior or posterior referencing, based on the morphology of posterior condyles. However, gaps are not quantified and are balanced on rotations set by a 4-in-1 jig, requiring extensive soft tissue releases. In cases of valgus deformity due to tibial origin, valgus cuts taken in conventional TKA are independent of tibial defect, and there are higher chances of misinterpreting distal femur cuts. Proximal tibial and distal femur cuts are made before gap balancing. In RATKA, bone cuts are made after predictive gap balancing, thus achieving adequate correction of deformity. In our study, we had no such difficulty, and gaps were balanced with external or internal rotation of components, depending on flexion gaps with minimal bone cuts and without the need for the release of lateral structures to achieve equal gaps.

There is an ongoing debate in the literature over PS and CR implants [2] or higher-constrained implants for deformity correction. Implant selection, in our study, was carried out pre-operatively based on the radiological assessment, clinical evaluation, and MAKO Planning software, and revised intraoperatively if needed. We found a healthy PCL in most of the osteoarthritis cases, where CR or CS implants were used, depending on flexion stability. In most of the rheumatoid cases where PCL was lax or friable, PS implants were used. In all cases, gaps were adequately balanced and either CR or PS implants were used. Using less constrained implants and achieving optimum gaps with minimal bone cuts preserves the bone stock and further aids in future revision surgery.

Compared to their predecessors, current semi-active robotic systems exhibit higher technical reliability and a lower risk of complications than conventional TKA [12]. Furthermore, the haptic feedback provides superior soft tissue protection than conventional TKA, which may account for better early functional outcomes. Our study had no major complications and median blood loss was 478.45 (176.25) mL during surgery. Similar results were reported by Kayani et al. in their prospective cohort of 40 cases undergoing RATKA and 40 cases undergoing conventional TKA [53]. The median operating time in their study was 68.0 and 61.2 minutes in the robotic group and conventional group, respectively, compared to a mean operating time of 70.17 minutes in our study. They found those who underwent RATKA had improved outcomes regarding post-operative pain, anesthesia requirement, and post-operative hemoglobin levels [53].

The use of RATKA in this study was associated with a significant improvement in deformity correction with a minimal need for soft tissue release. The primary surgeon had substantial experience in computer-assisted surgeries, encountered no challenges in adapting to the technology, and used a single approach in all cases. Post-operatively, patients recovered significantly in the first 6 weeks without any instability or implant loosening over the two-year follow-up. The mean valgus deformity correction achieved intraoperatively was 1.92 ± 0.77 in femur and 1.25 ± 0.53° in tibia with a mean of 5.83 ± 2.11° on the fifth day post-operatively, and 5.26 ± 1.53° in the sixth week, which had significant improvement. Distal fitting stems in the tibia were used only in patients with osteoporosis, however, stems were not used to correct the deformity.

#### Limitations

This prospective, single-surgeon study evaluated early two-year outcomes in a relatively small cohort of patients with grade 2 and 3 deformities. Therefore, longer duration of follow-up with a larger cohort of patients are needed. As this technology continues to expand and gain adoption across more hospitals and institutions, larger, prospective multicenter studies can be conducted, incorporating additional modalities for assessing patient satisfaction. Robotic technology is associated with substantial installation and maintenance costs, with further costs incurred for additional pre-operative imaging, increased operating times during the learning phase, training the surgical team, updating computer software and servicing contracts, and consumables. Many robotic devices are compatible with a limited number of implant designs.

## Conclusion

In our study, we found that RATKA is useful in correcting moderate to severe valgus knee deformity, without soft tissue release, with minimal bone resection and less constrained implants, resulting in improved early functional outcomes.

## Data Availability

Available upon request from the corresponding author.

## References

[1] MacDessi SJ, Griffiths-Jones W, Harris IA, et al. (2021) Coronal plane alignment of the knee (CPAK) classification: a new system for describing knee phenotypes. Bone Jt J 103-B, 329–337. 10.1302/0301-620X.103B2.BJJ-2020-1050.R1 PMC7954147 33517740

[2] Ranawat AS, Ranawat CS, Elkus M, et al. (2005) Total knee arthroplasty for severe valgus deformity. J Bone Jt Surg 87, 271–284. 10.2106/JBJS.E.00308 16140800

[3] Favorito PJ, Mihalko WM, Krackow KA (2002) Total knee arthroplasty in the valgus knee. J Am Acad Orthop Surg 10, 16–24. 11809047 10.5435/00124635-200201000-00004

[4] Rajgopal A, Dahiya V, Vasdev A, et al. (2011) Long-term results of total knee arthroplasty for valgus knees: soft-tissue release technique and implant selection. J Orthop Surg 19, 60–63. 10.1177/230949901101900114 21519079

[5] Xie K, Lyons ST (2017) Soft tissue releases in total knee arthroplasty for valgus deformities. J Arthroplasty 32, 1814–1818. 28236551 10.1016/j.arth.2017.01.024

[6] Krackow KA, Jones MM, Teeny SM, Hungerford DS (1991) Primary total knee arthroplasty in patients with fixed valgus deformity. Clin Orthop (278), 9–18. 1959292

[7] Lombardi AV, Dodds KL, Berend KR, et al. (2004) An algorithmic approach to total knee arthroplasty in the valgus knee. J Bone Jt Surg 86, 62–71. 10.2106/00004623-200412002-00010 15691110

[8] Mou P, Zeng Y, Yang J, et al. (2018) The effectiveness of medial femoral epicondyle up-sliding osteotomy to correct severe valgus deformity in primary total knee arthroplasty. J Arthroplasty 33, 2868–2874. 29805102 10.1016/j.arth.2018.04.045

[9] Karachalios T, Sarangi PP, Newman JH (1994) Severe varus and valgus deformities treated by total knee arthroplasty. J Bone Joint Surg Br 76-B, 938–942. 7983123

[10] Li H, Ponzio DY, Ong A, et al. (2020) Total knee arthroplasty for fixed valgus deformity correction using a modified lateral parapatellar approach. J Knee Surg 33, 372–377. 30727017 10.1055/s-0039-1677821

[11] Keblish PA (1991) The lateral approach to the valgus knee. Surgical technique and analysis of 53 cases with over two-year follow-up evaluation. Clin Orthop (271), 52–62. 1914314

[12] Tang Q, Shang P, Zheng G, et al. (2017) Extramedullary versus intramedullary femoral alignment technique in total knee arthroplasty: a meta-analysis of randomized controlled trials. J Orthop Surg 12, 82. 10.1186/s13018-017-0582-3 PMC5460512 28583144

[13] Charette RS, Sheth NP, Boettner F, et al. (2018) Femoral component sizing during total knee arthroplasty: anterior versus posterior referencing. JBJS Rev 6, e4. 10.2106/JBJS.RVW.17.00051 29337712

[14] Boyer B, Pailhé R, Ramdane N, et al. (2018) Under-corrected knees do not fail more than aligned knees at 8 years in fixed severe valgus total knee replacement. Knee Surg Sports Traumatol Arthrosc 26, 3386–3394. 29594324 10.1007/s00167-018-4906-6

[15] Alghamdi A, Rahmé M, Lavigne M, et al. (2014) Tibia valga morphology in osteoarthritic knees: importance of preoperative full limb radiographs in total knee arthroplasty. J Arthroplasty 29, 1671–1676. 24726171 10.1016/j.arth.2014.03.001

[16] Lange J, Haas SB (2017) Correcting severe valgus deformity: taking out the knock. Bone Jt J 99-B, 60–64. 10.1302/0301-620X.99B1.BJJ-2016-0340.R1 28042120

[17] Mullaji AB, Shetty GM (2010) Lateral epicondylar osteotomy using computer navigation in total knee arthroplasty for rigid valgus deformities. J Arthroplasty 25, 166–169. 19679436 10.1016/j.arth.2009.06.013

[18] Gehrke T, Kendoff D, Haasper C (2014) The role of hinges in primary total knee replacement. Bone Jt J 96-B, 93–95. 10.1302/0301-620X.96B11.34143 25381417

[19] Adravanti P, Vasta S (2017) Varus-valgus constrained implants in total knee arthroplasty: indications and technique. Acta Bio-Medica Atenei Parm 88, 112–117. 10.23750/abm.v88i2-S.6521 PMC6178999 28657572

[20] Alesi D, Meena A, Fratini S, et al. (2022) Total knee arthroplasty in valgus knee deformity: is it still a challenge in 2021? Musculoskelet Surg 106, 1–8. 33587251 10.1007/s12306-021-00695-x PMC8881420

[21] Koutserimpas C, Favroul C, Batailler C, et al. (2024) Is bicortical femoral pin insertion safe for image-based robotic knee arthroplasty surgery? A comparative complications analysis in 970 consecutive cases. J ISAKOS 10, 100317. 39251024 10.1016/j.jisako.2024.100317

[22] Paley D, Pfeil J (2000) Principles of deformity correction around the knee. Orthopade 29, 18–38. 10663243 10.1007/s001320050004

[23] Khlopas A, Sodhi N, Sultan AA, et al. (2018) Robotic arm-assisted total knee arthroplasty. J Arthroplasty 33, 2002–2006. 29506926 10.1016/j.arth.2018.01.060

[24] Shatrov J, Battelier C, Sappey-Marinier E, et al. (2022) Functional alignment philosophy in total knee arthroplasty – rationale and technique for the varus morphotype using a CT based robotic platform and individualized planning. SICOT-J 8, 11. 35363136 10.1051/sicotj/2022010 PMC8973302

[25] Deep K (2014) Collateral ligament laxity in knees: what is normal? Clin Orthop 472, 3426–3431. 25115587 10.1007/s11999-014-3865-6 PMC4182367

[26] Shatrov J, Foissey C, Kafelov M, et al. (2023) Functional alignment philosophy in total knee arthroplasty – rationale and technique for the valgus morphotype using an image based robotic platform and individualized planning. J Pers Med 13, 212. 36836446 10.3390/jpm13020212 PMC9961945

[27] Kayani B, Konan S, Pietrzak JRT, et al. (2018) The learning curve associated with robotic-arm assisted unicompartmental knee arthroplasty: a prospective cohort study. Bone Jt J 100-B, 1033–1042. 10.1302/0301-620X.100B8.BJJ-2018-0040.R1 30062950

[28] Bautista M, Manrique J, Hozack WJ (2019) Robotics in total knee arthroplasty. J Knee Surg 32, 600–606. 30822790 10.1055/s-0039-1681053

[29] Peng L, Luo Y, Liu J, Li Z (2020) The efficacy of patellar denervation with electrocautery after total knee replacement: a meta-analysis of randomized controlled trials. Int J Surg 78, 126–137. 32335235 10.1016/j.ijsu.2020.04.049

[30] Gustke KA, Golladay GJ, Roche MW, et al. (2014) A new method for defining balance. J Arthroplasty 29, 955–960. 24269069 10.1016/j.arth.2013.10.020

[31] Singh Sidhu GA, Marya SKS, Singh C, et al. (2021) Efficacy of topical versus intravenous tranexamic acid in bilateral total knee arthroplasty. J Arthrosc Jt Surg 8, 346–349.

[32] Rorabeck CH, Taylor JW (1999) Classification of periprosthetic fractures complicating total knee arthroplasty. Orthop Clin North Am 30, 209–214. 10196422 10.1016/s0030-5898(05)70075-4

[33] Stehlík J, Musil D, Held M, Stárek M (2006) Z-plasty for valgus deformity in total knee arthroplasty. Acta Chir Orthop Traumatol Cech 73, 169–175. 16846562

[34] Miyasaka KC, Ranawat CS, Mullaji A (1997) 10- to 20-year followup of total knee arthroplasty for valgus deformities. Clin Orthop (345), 29–37. 9418618

[35] Easley ME, Insall JN, Scuderi GR, Bullek DD (2000) Primary constrained condylar knee arthroplasty for the arthritic valgus knee. Clin Orthop 380, 58–64. 10.1097/00003086-200011000-00008 11064973

[36] Batailler C, Lording T, Libert T, et al. (2025) Achieving better clinical outcomes after total knee arthroplasty in knees with valgus deformity: the role of alignment strategies. J Bone Jt Surg 107(2), 152–162. 10.2106/JBJS.24.00207 39591439

[37] Parratte S, Pagnano MW, Trousdale RT, Berry DJ (2010) Effect of postoperative mechanical axis alignment on the fifteen-year survival of modern, cemented total knee replacements. J Bone Joint Surg Am 92, 2143–2149. 20844155 10.2106/JBJS.I.01398

[38] Rivière C, Iranpour F, Auvinet E, et al. (2017) Alignment options for total knee arthroplasty: a systematic review. Orthop Traumatol Surg Res 103, 1047–1056. 28864235 10.1016/j.otsr.2017.07.010

[39] Cortina G, Za P, Papalia GF, et al. (2023) Restricted kinematic alignment is clinically non-inferior to mechanical alignment in the short and mid-term: a systematic review. The Knee 45, 137–146. 37925804 10.1016/j.knee.2023.10.003

[40] Lustig S, Sappey-Marinier E, Fary C, et al. (2021) Personalized alignment in total knee arthroplasty: current concepts. SICOT-J 7, 19. 33812467 10.1051/sicotj/2021021 PMC8019550

[41] Karasavvidis T, Pagan Moldenhauer CA, Lustig S, et al. (2023) Definitions and consequences of current alignment techniques and phenotypes in total knee arthroplasty (TKA) – there is no winner yet. J Exp Orthop 10, 120. 37991599 10.1186/s40634-023-00697-7 PMC10665290

[42] Oussedik S, Abdel MP, Victor J, et al. (2020) Alignment in total knee arthroplasty. Bone Jt J 102-B, 276–279. 10.1302/0301-620X.102B3.BJJ-2019-1729 32114811

[43] Bonner TJ, Eardley WGP, Patterson P, Gregg PJ (2011) The effect of post-operative mechanical axis alignment on the survival of primary total knee replacements after a follow-up of 15 years. J Bone Joint Surg Br 93-B, 1217–1222. 10.1302/0301-620X.93B9.26573 21911533

[44] Nam D, Nunley RM, Barrack RL (2014) Patient dissatisfaction following total knee replacement: a growing concern? Bone Jt J 96-B, 96–100. 10.1302/0301-620X.96B11.34152 25381418

[45] Kuo AW, Chen DB, Wood J, MacDessi SJ (2020) Modern total knee arthroplasty designs do not reliably replicate anterior femoral morphology. Knee Surg Sports Traumatol Arthrosc 28, 2808–2815. 31352496 10.1007/s00167-019-05610-3

[46] Shatrov J, Coulin B, Batailler C, et al. (2023) Alignment philosophy influences trochlea recreation in total knee arthroplasty: a comparative study using image-based robotic technology. Int Orthop 47, 329–341. 36112197 10.1007/s00264-022-05570-3 PMC9877070

[47] Dretakis K, Koutserimpas C (2024) Pitfalls with the MAKO robotic-arm-assisted total knee arthroplasty. Medicina (Mex) 60, 262. 10.3390/medicina60020262 PMC10890000 38399549

[48] Siddiqi A, Smith T, McPhilemy JJ, et al. (2020) Soft-tissue balancing technology for total knee arthroplasty. JBJS Rev 8, e0050. 31899697 10.2106/JBJS.RVW.19.00050

[49] Rossi SMP, Sangaletti R, Andriollo L, et al. (2024) The use of a modern robotic system for the treatment of severe knee deformities. Technol Health Care 32, 3737–3746. 38251078 10.3233/THC-231261

[50] Marchand R, Sodhi N, Khlopas A, et al. (2018) Coronal correction for severe deformity using robotic-assisted total knee arthroplasty. J Knee Surg 31, 2–5. 29179223 10.1055/s-0037-1608840

[51] Pagoti R, O’Brien S, Doran E, Beverland D (2017) Unconstrained total knee arthroplasty in significant valgus deformity: a modified surgical technique to balance the knee and avoid instability. Knee Surg Sports Traumatol Arthrosc 25, 2825–2834. 26615591 10.1007/s00167-015-3881-4

[52] Song E-K, Seon J-K, Yim J-H, et al. (2013) Robotic-assisted TKA reduces postoperative alignment outliers and improves gap balance compared to conventional TKA. Clin Orthop 471, 118–126. 22669549 10.1007/s11999-012-2407-3 PMC3528918

[53] Kayani B, Konan S, Tahmassebi J, et al. (2018) Robotic-arm assisted total knee arthroplasty is associated with improved early functional recovery and reduced time to hospital discharge compared with conventional jig-based total knee arthroplasty: a prospective cohort study. Bone Jt J 100-B, 930–937. 10.1302/0301-620X.100B7.BJJ-2017-1449.R1 PMC6413767 29954217

